# Trial-by-Trial Adaptation of Movements during Mental Practice under Force Field

**DOI:** 10.1155/2013/109497

**Published:** 2013-05-07

**Authors:** Muhammad Nabeel Anwar, Salman Hameed Khan

**Affiliations:** Human Systems Laboratory (HSL), Department of Biomedical Engineering and Sciences, School of Mechanical & Manufacturing Engineering, National University of Sciences & Technology, Islamabad 44000, Pakistan

## Abstract

Human nervous system tries to minimize the effect of any external perturbing force by bringing modifications in the internal model. These modifications affect the subsequent motor commands generated by the nervous system. Adaptive compensation along with the appropriate modifications of internal model helps in reducing human movement errors. In the current study, we studied how motor imagery influences trial-to-trial learning in a robot-based adaptation task. Two groups of subjects performed reaching movements with or without motor imagery in a velocity-dependent force field. The results show that reaching movements performed with motor imagery have relatively a more focused generalization pattern and a higher learning rate in training direction.

## 1. Introduction

Mental simulation of various actions can be used as a tool for studying theoretical concepts about cognitive neuroscience. Motor imagery, a subcategory of mental simulation, is an internal reproduction of a specific motor action without any overt motor output and is widely used for improving the motor performance. In relation to it, the underlying neurological mechanisms activated by mentally rehearsing motor actions are quite similar to the ones activated during actual physical movements [1]. There is a high overlap between the active brain regions of subjects undergoing movement execution and the movement imagination [2]. It provides the idea that motor imagery might help the CNS in the learning process and can be used in conjunction with physical training to improve motor performance [2–5]. As an example, it is used for improving the performance of athletes and sports men [6]; experienced musicians have used motor imagery for improving coordination between complex spatial and timing components of a musical composition [2]. It is also used, for speeding up the recovery process of stroke patients and neurological rehabilitation [7], for motion accuracy, and for adaptation to the changing dynamics and arm kinematics [5, 8].

In the current study we consider a task in which subjects make series of reaching movements in the presence of external dynamics, that is, an externally imposed force field from a mechanical robot. The force field introduces significant errors in contrast to the movements that take place in the absence of any external force field. These errors gradually fade out with practice as the nervous system adapts to the newly imposed dynamics; this recovery of performance is “*motor adaptation*” [9]. The force field is switched off unexpectedly in some trials during adaptation; these trails are termed as “*catch trials”* and they help in investigating the properties of internal model that human nervous system updates to predict and neutralize the error. The trajectories formed during the catch trials are quite similar in shape but opposite in direction to the trajectories that are observed at the sudden introduction of force field (also termed as “*after effects”*). This supports the notion that model-based motor commands are generated by central nervous system (CNS). There are predominantly two modes in human motor control mechanism: feedback and feedforward. During the early learning stage, internal model is evolved and learning is achieved through sensorimotor feedback mechanism. After sufficient practice, motor systems adapt with external environment and operate autonomously in feedforward mode; this is called as “late learning” [10].

Human brain formulates internal model in such a way that motor learning in one direction has a positive impact on learning in other adjacent directions. This effect gradually decreases as the difference in directions increases. The ability to apply what has been learned in one context to other contexts is termed as the “*generalization”* of motor learning. When generalization increases learning in some contexts, it is called as “*transfer.”* In some contexts, generalization diminishes learning and it is said to be causing an “*interference”* [11]. It shows that the model evolved by human nervous system learns beyond the boundaries of training data and its output is broadly adapted across the state space of motor commands [9]. In the contexts where generalization is detrimental, it is usually due to the large alteration in the learning problem associated with comparatively small contextual changes. This is relevant to our experiment where a large change in direction, that is, around 135° to 180°, has an associated small change in context. This is also true in general; for example, driving car in the reverse direction or counting backward is difficult as compared to normal routine.

The current work is focused on how CNS learns to control and compensate errors in imagined reaching movements and how an error experienced in one direction can affect the reaching movements in other directions, with or without motor imagery. In other words, an investigation is made to answer how mental practice affects the generalization pattern of internal learning model developed by CNS. Up to the best of our knowledge, the relation between generalization and motor imagery in reaching movements has not been studied explicitly. By motor imagery, we mean that the individual subjects imagine the subsequent movement before actually performing it (MI group). The group of subjects without any conscious intent before starting movement or has not mentally rehearsed the upcoming movement constitutes the no motor imagery group (No-MI group).

At this stage we develop our initial hypothesis as follows.

1. The motor imagery affects the generalization function in such a way that it transfers the learning in nearby directions.
2. The group of subjects who rehearsed the task mentally prior to their physical action will have a high learning rate in the direction of training and associated directions.
3. The group of subjects who rehearsed the task mentally prior to the physical action will have a more focused generalization pattern with respect to the No-MI group.

The composition of the remaining paper is as follows.  Section 2 describes the related work. Methods and materials are explained in Section 3. Results are outlined in Section 4. The conclusion and future work are included in Section 5.

## 2. Related Work

Mussa-Ivaldi and  Bizzi studied the possible ways in which the information about force field dynamics was perceived by the CNS. Finding the movement path based on perception of force field is a complex inverse dynamics problem, and brain forms an internal model composed of motor primitives to solve this inverse problem. This internal model is updated regularly to conform with the ever-changing environmental and physical dynamics [12]. Robotic manipulandum systems are widely used to study the underlying dynamics of motor commands issued by CNS [13].

Previous studies suggest that motor imagery has a constructive effect on the human motor performance. It has been argued that the covert mental practice is a cost effective, easily accessible strategy to improve motor performance of affected body parts after stroke [14]. Gentili et al. have studied the associated question of how imagination and mental execution of physical activities can help in learning process. It is found that although subjects with physical training (without imagery) have good learning rate than the subjects undergoing mental training (without any sensorimotor feedback), yet the movement rhythms and adaptation rates were identical. Authors proposed that the internal forward model of human brain provides state estimation to improve motor performance during imagery [5].

## 3. Materials and Methods

### 3.1. Experimental Setup

**Figure 1:**
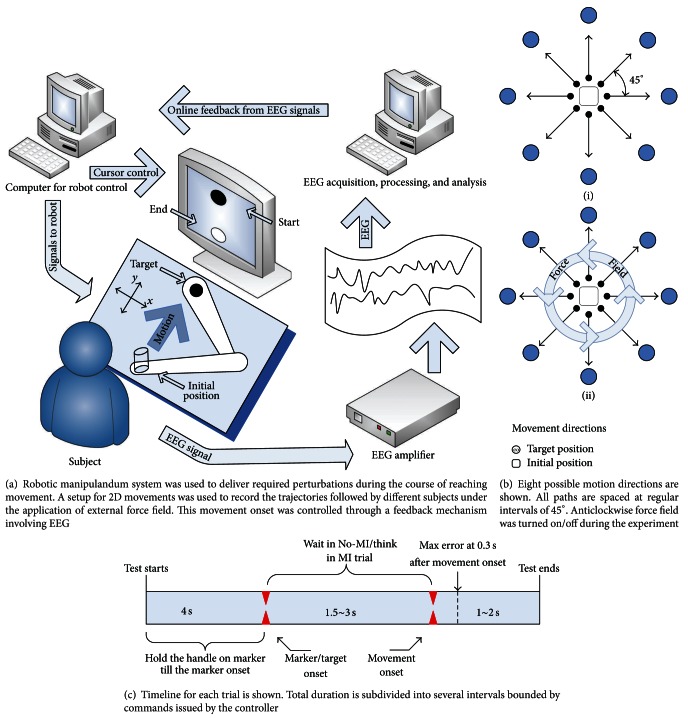
Experimental setup and trial protocol.

We considered a behavioral task for studying the effect of motor imagery on trial-by-trial motor learning. The subjects performed center out reaching movements by using a robotic manipulandum. An external force field was generated by the robotic manipulandum for desired perturbations in a plane during the movements. The subjects, then, had to adapt to the new environment. This helped in studying the adaptive capabilities of human motor system Figure 1(a).

The experimental setup shown in Figure 1(a) was the same as [8]. In this setup the *Braccio di Ferro* robot (see [15] for details) was used to generate the forces and record the motion paths. The plane of motion was restricted to only two dimensions for the ease of analysis. Fourteen-channel EEG was recorded using gold cup electrodes (g.EEGcap g.tec, Guger Technologies OEG, Graz, Austria). The electrodes were placed at central locations (C3, C1, Cz, C2, and C4), frontal locations (F3, Fz, and F4), parietal locations (P3, Pz, and P4), and temporal locations (T3 and T4) by adapting international 10–20 electrode placement system. Left earlobe and right earlobe were used as reference and ground, respectively. Analogue EEG signals were amplified and band-pass filtered (0.1–100 Hz) by the EEG amplifier (g.BSamp g.tec, Guger Technologies OEG, Austria). The signals were then sampled at 256 Hz (NIDAQ 6040-E) and were stored for later offline analysis. The online feedback was provided by a software application based on BCI2000 [16].

#### 3.1.1. Subjects

Total 12 subjects participated in this experiment. Eleven of the subjects were right handed, while one left handed subject was present. Before undertaking the experiment, a screening process was performed in which EEG patterns of all subjects were analyzed. During this process, each subject was asked to rest for 3 seconds (base line) followed by imagining hand movements for 2 seconds, and a total of 96 trials were conducted in this way. Then, for each subject, we identified the spectral bandwidth and the electrode locations that correlated most with the motor imagery. The most responsive spectral bandwidth and electrode locations were then used for online feedback. We also calculated the “coefficient of determination” for each subject. It acts as a measure to determine the quality of human intention that can be inferred from the EEG signal. It is expressed as a correlation coefficient defined over a bivariate signal composed of EEG signal *x* during motor imagery and a task condition signal *y* that consists of EEG signal during rest period:

(1) r2=σ(x,y)2σ2(x)·σ2(y),

where *r* ^2^ value was calculated from each electrode. After screening, the subjects were randomly assigned to two experimental protocols as folows: “with imagery” (6 subjects, 1 M and 5 F, mean age 23 ± 1.5 years) and “no imagery” (6 subjects, 3 M and 3 F, mean age 25 ± 2.8 years).

### 3.2. Experimental Procedures

The subjects sat on a chair in front of the manipulandum. The height and position of the seat were adjusted so that the arm could be kept horizontally at shoulder level pointing towards the center of the work space. In normal position, the elbow and the shoulder joints were flexed about 90° and 45°, respectively. The experimental protocol was displayed to the subjects on a 19′′ LCD computer screen placed about 1 m away at eye level. The subjects performed 10 cm reaching movements with dominant hand. The targets were displayed on a black background as white circles of 1 cm diameter appeared at one of the eight random locations (0°, 45°, 90°, 135°, 180°, 225°, 270°, and 315°). The current position of the hand along with target was continuously displayed on the computer screen. 

The experiment was organized into sets; each set consisted of a sequence of 48 target presentations, with target appeared at 8 different positions, 6 times each. Every set lasted for approximately 7 ~ 8 minutes, and the subjects were allowed to take rest between sets. Each movement started from the center of the work space. In order to initiate a movement, the subject had to hold the cue at the starting point (initial position of the target). Once the cue is in center of the target, the target changed its color to gray; after 4 s it shifted to one of the eight random outer positions and turn into red. At this point, the “imagery” group subjects were required to “imagine” the hand movement toward the target. EEG signals were continuously recorded and after every 300 ms a spectral estimate in the most responsive frequency band was calculated. This value was compared with the threshold value to detect the presence/absence of event-related EEG desynchronisation (ERD). The binary signal was transmitted to the robot and used for changing the color of the target, that is, red to yellow to green. A “go” signal is then generated (target color turning into green), indicating that the actual movement could start. This signal can only be generated if either of the following conditions was fulfilled:

1. the subject successfully generated 5 ERDs or
2. the 3 sec time limit of waiting was reached.

In the “no imagery” experiments, only condition (2) was applied and the subjects had to wait for 1.5 to 3 sec randomly between target appearance and the “go” signal. On “go” signal, the subjects were required to move as fast and as accurate as possible. Subjects were encouraged to keep an approximately constant movement timings and to avoid eye blinking and head movements or throat clearing during the imagery and movement phase. The next trial started as soon the subject placed the cursor inside the target at the central initial position.

Movements were performed under three different conditions: (i) null field (robot generated no force, 5 target sets); (ii) force field (velocity dependent force field was turned on, 5 target sets); (iii) after-effect (no field again, 2 target sets). During force field trials, the robot generated a viscous curl field that perturbed the reaching movements. The force field was perpendicular to the instantaneous hand velocity vector with magnitude proportional to the velocity

(2) F=B·ν,

where,

(3) B=[0−bb0]N×sm−1,

where the viscous coefficient *b* is 12 N · m^−1^ · s^−1^. The hand velocity vector (and its subsequent derivatives) was estimated online by means of a numerical differentiation technique. During the field sets, “catch trials” were inserted in which the force field was unexpectedly turned off. The probability of occurrence of one catch trial was set to 1/6, which corresponds to one catch trial per direction per set.

### 3.3. Data Analysis

#### 3.3.1. Screening

During screening phase, the recorded EEG data was arranged into 1 s long epochs and mean was removed. A 20th-order autoregressive model was used for estimating the power spectral density. The spectrum was calculated from 0 Hz to 40 Hz at every 0.2 Hz, and then spectral average was made into 2 Hz bins for 96 hand imagery trials and compared them with the rest period. The averaged spectral change (spectra at rest condition minus spectra during imagery) was also estimated during the screening process. Screening gave an overview about the most responsive electrode and the maximum change in the ERD. This information was the basis of online feedback.

#### 3.3.2. Online Feedback

EEG signals were recorded in 300 ms blocks, and for each block the software application estimated the power spectral density. The online ERD detection threshold was set at the 80% of the averaged spectral change from the base measurement during the rest period. Thus, for each subject the threshold was different and it was 80% of the maximum spectral change he/she could produce. As a result, a binary signal, that is, 1 (presence of a ERD) or a 0 (no ERD) was generated after every 300 ms and was used to change the color of the target from red to yellow to green.

#### 3.3.3. Familiarization Session

For each subject, we tested the error measurements for normal distribution using Shapiro-Wilk, Kolmogorov-Smirnov, and Lilliefors tests. It turned out that the distributions were normal (*P* ≤ 0.01). Equivalent variances were tested using Hartley, Cochran, and Bartlett tests.

#### 3.3.4. Adaptation Session

Hand trajectories were sampled at 100 Hz. The *x* and *y* components were smoothed with a 6th-order Savitzky-Golay filter (window size 270 ms, equivalent cut-off frequency of around 7 Hz). The first three-time derivative was estimated for the following indicators of motor performance

*Aiming Error.* Aiming error provides angular difference between the required target direction and the actual hand movement direction in the early phase of the movement, that is, 300 ms from movement onset. This error provides information about the lateral deviation and is used as a general measure of curvature.

*Learning Index*. The learning process was quantified by using an indicator similar to that proposed by [17]. This measure is independent of the magnitude of force field and other user-specific parameters such as the net compliance of the arm:

(4) Ilearning=−yc|yc−yf|,

where *y*_*f*_ and *y*_*c*_ are the 300 ms aiming errors in the field and catch trials, respectively. Both error measures were adjusted for any bias present in the last null field set. Therefore, errors were always referred to change from errors in the null set.

### 3.4. State Space Modeling

Internal model developed by brain is composed of a set of primitives that translate desired movement trajectories into required motor commands. In an event of external perturbation, motor commands are issued to minimize its effects. The forces produced as a result can be expressed in terms of desired position and velocity primitive functions *g*_*j*_ [18]:

(5) 𝒪=WT·g(x,dxdt) ∣ g=[g1,g2,…,gj]T,

*W* ^*T*^ is the experience dependent weighted matrix which is adjusted according to

(6) ΔWi=−η·g(xi,dxidt).

The shape of the primitives in the above equations can be found out by fitting a linear state space model over experimental data. Such a fit is possible as explained in [17, 19]. Although, various types of models can be used for dynamic system modeling, we used prediction error estimate method (PEM) to identify a structured linear state space model. PEM algorithm is quite similar to maximum likelihood estimation used in time series analysis [20].

Let us suppose that we have eight dimensional input force field signal denoted by *f*(*n*), which triggers maximum speed path error of *e*(*n*) with sample number *n*. If we have data upto *N*th sample a set of input output pairs can be defined as,

(7) 𝒳={f(n),e(n)} ∣ 1≤n≤N.

Here, the input *f*(*n*) is dependent on the trial which may be a force field trial (FF), simple null field trial (NF) or null field catch trial (C) after the removal of force field;

(8) t∈{C,NF,FF}.

Linear state space model can be represented as a predictor model that estimates (*N* + 1)th output sample:

(9) e^([N+1] ∣ N;φ)=ℱ(𝒳N,φ).

Using an iterative procedure, an estimate of parameter vector ${\hat{e}}_{N+1}$ is generated for (*N* + 1)th output. ${\hat{e}}_{N+1}$ depends both on samples from 1 ⋯ *N* and parameter vector *φ*. *φ* represents the parametrization, and *ℱ*(·) is the function defined on observed data [20]:

(10) ℱ(𝒳N,φ)=He(q,φ)e(n)+Hf(q,φ)f(n)=∑k  =  1Nhe(k)e(n−k)+∑k=1Nhf(k)f(n−k),

where *q* is a shift operator and *H*_*e*_ and *H*_*f*_ are the linear time or shift invariant filters which we will specify in a further discussion. State space equations are given by

(11) x˙(n+1)=A(φ)x(n)+B(φ)f(n),e(n)=C(φ)x(n)+Df(n).

The linear state space model is estimated based on the assumption that the data has been generated according to (11). PEM tries to minimize a weighted norm of estimation error. In our case, where there is only one output, this cost function *ξ*_*N*_(·) is given by

(12) ξN(R,S)=1S2(q,φ)∑t=1NΔΔT,Δ=e(n)−e^(n ∣ φ),

where $\hat{e}(n|\varphi )$ is the output estimate of model, and PEM produces an output which is optimal in least squares sense. *N* is the number of data values of errors during hand-reaching experiments. In the cost function estimated output is supposed to be:

(13) e^(n ∣ φ)=R(q,φ)·f(n),

where *f*(*n*) is the input of the model. Here, *R*(*q*, *φ*) and *S*(*q*, *φ*) are the matrices that can be described in terms of state space matrices. In turn, they define filters as follows:

(14) He(q,φ)=[I−S−1(q,φ)],Hf(q,φ)=S−1(q,φ)  R(q,φ).

PEM is a fast algorithm and has similar merits as that of maximum likelihood estimation. However, it requires accurate parameterization and may get stuck in local minima. The initial parameters were estimated using a numerical algorithm for subspace state space system identification that projects both input and output data to find optimal state sequence (N4SID Algorithm by van Overschee and de Moor [21]). These sequences can be interpreted in terms of states of a parallel bank of Kalman filters. By using this interpretation, state space system matrices can be easily determined from the given data with no requirement of providing parameterization for nonzero initial conditions [21]. This algorithm uses QR decomposition and singular value decomposition. Thus, it is numerically stable and always converges to a finite value. With these benefits, we used it for finding an initial estimate of state space matrices of linear model.

In our experiments, the force field magnitude and direction (anticlockwise) were kept constant and its presence or absence was recorded using normalized integers;

(15) f(n)={+1,if  t∈{C,NF},−1,  if  t∈{FF}.

On each sampled input, a value of −1 indicates the presence of force field while a value of 1 indicates a catch trial or null field. Similar discrete scalar representation of force field magnitude was adopted by Thoroughman and Shadmehr [18] and Smith and Shadmehr [17].

Instead of using coordinate information in maximum errors, we used the relationship between actual arm compliance and the angular error. The details of the derivation can be found in [17]. In general, the two-dimensional compliance matrix is given by

(16) [xy]=[D11D12D21D22][fxfy].

This two-dimensional compliance matrix can be transformed to one-dimensional oppositional compliance having a value in each direction of motion. The magnitude of one-dimensional compliance matrix depends on direction of force and three parameters *D*_11_, *D*_22_, *D*_21_, and *D*_12_, see [17] for details. Briefly,

(17) D1(ϱ)=D11+D222+D11−D222cos⁡(2ϱ)+D12+D212sin(2ϱ).

We parameterized *D* matrix of the state space model with the value of *D*_1_(*ϱ*).

### 3.5. Measuring Goodness of Fit

**Figure 2:**
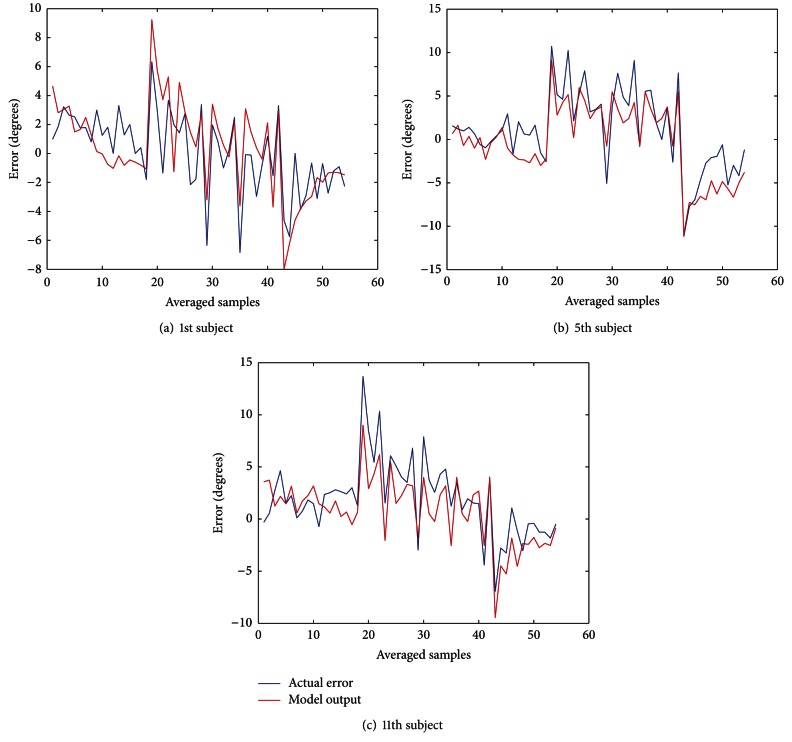
Time series of actual movement errors and the corresponding model predictions are shown. The changing trend of model output conforms with the actual movement errors. For the sake of clarity, the values are plotted after averaging every 5 samples.

We also compared the variances of estimated output and the actual errors (see Figure 2) to account for the goodness of fit of our model. We defined our goodness of measure by *δ* [19] as follows:

(18) δ=1−∑n=1N||e^(n)−e0(n)||∑n=1N||e(n)−e0(n)||,

where  *e*_0_(*n*) is a *baseline* model obtained by setting matrices *B* and *D* to zero. In (11),

(19) x˙(n+1)=A(φ)x(n),e0(n)=C(φ)x(n).

For our experiments, the model fit was reasonably good in subjects data (with a mean *μ*≅82% and standard deviation *σ*≅0.078). In comparison, Krakauer et al. in [11] do not report the error numerically, although authors state that model parameters are chosen such that the mean square error between model prediction and actual experimental data is minimized. Thoroughman and Shadmehr have reported 60% model fitness in their experiments related to human motor learning [18]. Donchin et al. have documented percentage deviation in model and actual output to be 77% [19]. Scheidt et al. report the variance accounted for (VAF) of 84% as the measure of error of their model [22]. In the nutshell, our model fit is competitive with the results reported in previous model-based studies.

## 4. Results and Discussion

Figure 4 shows the group averaged ERD patterns during the online feedback. The subjects in No-MI group were waiting for the “Go” signal, while the subjects from MI group were imagining upcoming movement. Both groups showed ERDs, however, ERDs in MI group were more prominent.

From the model parameters it is found that the directional changes of equal magnitude have nearly same estimated values. For the sake of convenience we reduced the number of free parameters to 5 by averaging the parameters on same directional difference values. Let *B*_*j*_^*i*^ be the vector in direction *j* for user *i*. Thus, for each direction *j*, we can formulate a matrix *𝒟*_*j*_ for both MI and No-MI subjects as follows:

(20) 𝒟j=[Bj1,Bj2,…,Bj6]T.

A new matrix *ℳ* can be defined over to reduce the free parameters to 5 by averaging the values on similar distance from peak learning rate. Each column *ℳ*_*l*_ can be defined by vectors

(21) ℳl=Bj+ki+Bj+ki2 0≤k≤4,

where *j* + *k* wraps around in an event of dimension outflow.

### 4.1. Statistical Analysis of Model Parameters

The variables were found to be normally distributed when Shapiro-Wilk *W*-test was applied. Setting the null hypothesis that the variables came from a normal distribution, we found the *P* values which were greater than the threshold of 0.05 in all cases. Next, we made comparison of relationships between variables (the learning rate in various directions) belonging to MI and No-MI groups by a parametric statistical test named *t*-test. We found that the MI group has higher learning rate than the corresponding No-MI group in all directions. In direction 0°, the learning rate of MI group is 0.203 ± 0.019 and for No-MI group's  0.175 ± 0.024 (*P* = 0.046). In direction 45°, MI group: 0.175 ± 0.026; No-MI group: 0.155 ± 0.023 (*P* = 0.05). In direction 90°, MI group has learning rate 0.184 ± 0.015 in contrast to No-MI group's 0.145 ± 0.031 (*P* = 0.020). In direction 135°, MI group: 0.195 ± 0.016; No-MI group: 0.155 ± 0.031 (*P* = 0.023). In direction 180°, MI group has learning rate 0.224 ± 0.019 in contrast to No-MI group 0.168 ± 0.033 (*P* = 0.005). In direction 225°, MI group: 0.197 ± 0.019, while No-MI group: 0.161 ± 0.030 (*P* = 0.039). In direction 270°, MI group has learning rate 0.189 ± 0.024, while No-MI group 0.148 ± 0.025 (*P* = 0.016). In direction 315°, MI group has learning rate of  0.207 ± 0.029 and No-MI group of 0.165 ± 0.022 (*P* = 0.021). See Figure 5 for a plot of comparison between MI and No-MI groups.

The effect of learning in one direction on immediate next direction was also analyzed. Along 0° the transfer of learning rate for MI group is = 0.112 ± 0.007, and for No-MI group it was  0.08 ± 0.009 (*P* = 0.037). In direction 45°, MI group has transfer of learning rate 0.125 ± 0.009 in contrast to No-MI group 0.092 ± 0.018 (*P* = 0.021). In direction 90°; MI group: 0.104 ± 0.006 and No-MI group: 0.087 ± 0.012 (*P* = 0.039). In direction 135°; MI group: 0.131 ± 0.004 in contrast to No-MI group: 0.077 ± 0.006 (*P* = 0.019). In direction 180°; MI group: 0.122 ± 0.009 and No-MI group: 0.087 ± 0.014 (*P* = 0.025). In direction 225°; MI group: 0.099 ± 0.011 and No-MI group: 0.081 ± 0.023 (*P* = 0.055). In direction 270°; MI group: 0.114 ± 0.012 and No-MI group: 0.100 ± 0.016 (*P* = 0.032). In direction 315°; MI group: 0.108 ± 0.019 in contrast to No-MI group: 0.072 ± 0.022 (*P* = 0.041). See Figure 6 for a comparison between MI and No-MI groups. 

Next, we studied the generalization patterns for MI and No-MI groups averaged over all the subjects in each group, see Figure 7. Student's *t*-test was performed to account for the significance level of results. Variables were found to be significantly different for MI and No-MI groups with *P* values < 0.05 in all directions except in direction 90°. The mean values of learning rate of all users with associated standard deviation are shown in Figure 7. It must be noted that the absolute values of learning rates in directions 135° and 180° are shown for the sake of easy comparison with learning rates in other directions.

### 4.2. Insights

**Figure 3:**
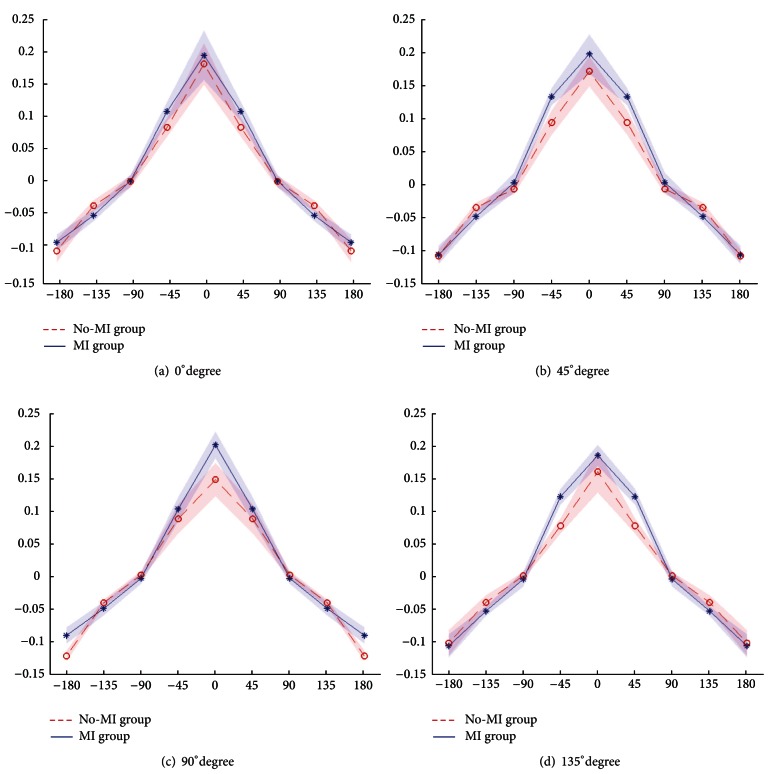
Generalization patterns in 4 directions are shown. Free parameters are reduced to 5 by averaging the parameters existing at same directional difference values. Shaded regions show the deviation in parameter values across all subjects.

**Figure 4:**
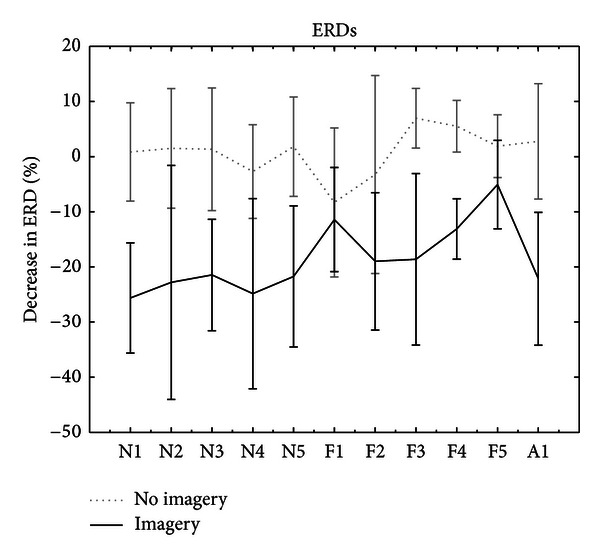
Figure shows averaged ERD patterns in both MI (in black) and No-MI (in dotted gray) groups. The ERD was calculated for each direction and was averaged within the sets. MI group has shown more prominent ERDs during imagery time.

**Figure 5:**
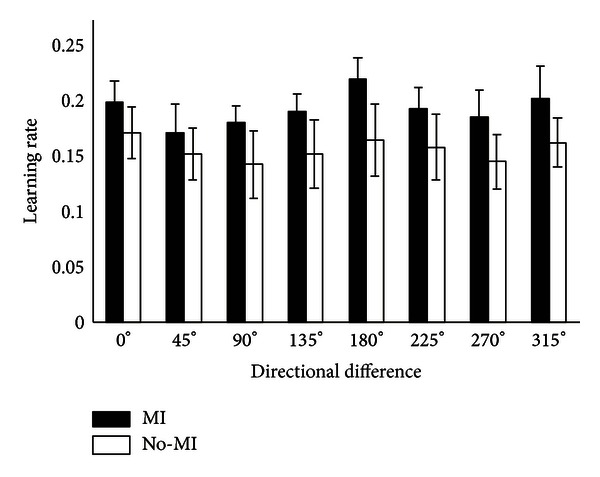
Motor learning rate in all directions is shown. The solid bars represent mean values while corresponding standard deviation values are represented by the limits put on bars.

**Figure 6:**
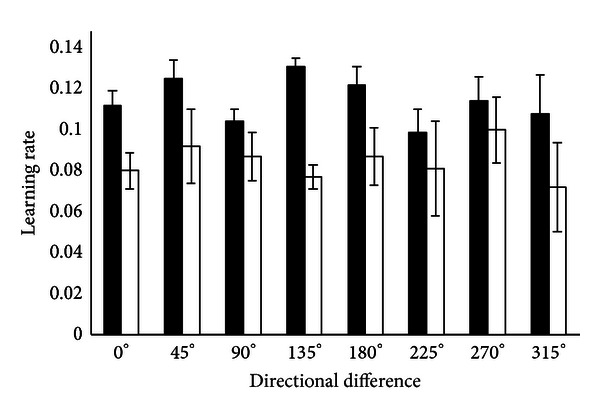
Motor learning rate that is, transferred in adjacent direction is shown. The impact of motor learning on immediate next direction (with 45° difference) is averaged across all subjects.

**Figure 7:**
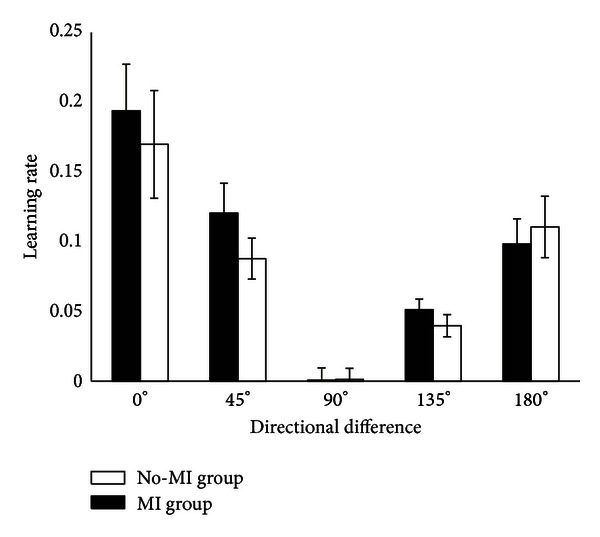
Averaged generalization pattern in all possible directional differences for MI and No-MI groups. Solid bars show the mean values, while the deviation is represented by the limits put on bars.

**Figure 8:**
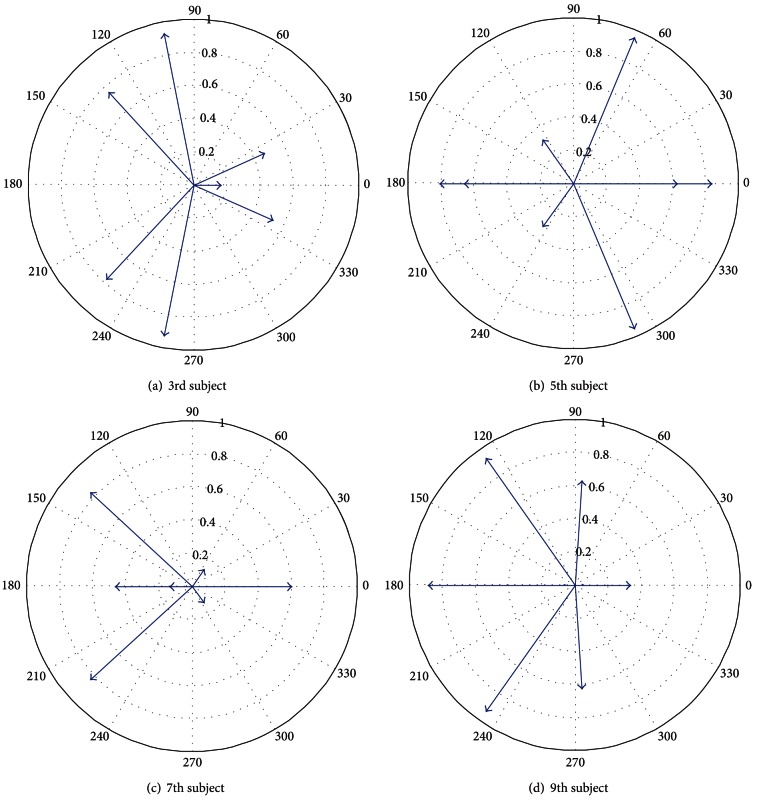
Polar plots for the eigenvalues of all odd subjects are shown. These plots signify that the model built for each subject is stable with the eigenvalues lying inside the unit circle.

Generalization patterns along directions  0°, 45°, 90°, and 135° degrees are shown in Figure 3. Also from Figures 5, 6, and 7 it is evident that the subjects with motor imagery have higher learning rates as compared to those of No-MI subjects. Mental rehearsal has focused the learning rate in one particular direction (the one in which training is performed). Generally, in both groups the trial-to-trial transfer of learning has decreased as the directional difference increase, but the transfer rate is higher in MI group. In case of 90° directional difference, the averaged generalization pattern shows that the mean and SD for both MI ad No-MI are not significantly different. This can be attributed to the fact that the perpendicular motion is unique and not much difficult to perform. Thus the learning transfer is less as compared to other direction. All the models were found to have a good fit and stable eigenvalues as shown in Figure 8. 

## 5. Conclusion

In this study we compared the performance of two groups of subjects (MI and No-MI) in a center out-reaching movement task under a force field. The small number of subjects in both groups is a limitation of this study and suggests the need for caution in the interpretation of our results. However, this study helped us to investigate the trial to trial effect of motor imagery on learning. It turned out that our initial three hypotheses were true (see Section 1). MI group has a higher learning rate and transfer of learning as compared to No-MI group and has a more focused generalization pattern. These results show positive influence of motor imagery and suggest that motor learning can be facilitated by mentally rehearsing the upcoming movement and could be used to increase the rate of adaptation.
